# Achieving Both Ultrahigh Electrical Conductivity and Mechanical Modulus of Carbon Films: Templating‐Coalescing Behavior of Single‐Walled Carbon Nanotube in Polyacrylonitrile

**DOI:** 10.1002/advs.202205924

**Published:** 2023-01-22

**Authors:** Jung‐Eun Lee, Jung Hoon Kim, Joong Tark Han, Han Gi Chae, Youngho Eom

**Affiliations:** ^1^ Department of Materials Science and Engineering Ulsan National Institute of Science and Technology (UNIST) Ulsan 44919 Republic of Korea; ^2^ Nano Hybrid Technology Research Center Korea Electrotechnology Research Institute (KERI) Changwon‐si Gyeongsangnam‐do 51543 Republic of Korea; ^3^ Electrical Functional Material Engineering Korea University of Science and Technology (UST) Changwon‐si Gyeongsangnam‐do 51543 Republic of Korea; ^4^ Department of Polymer Engineering Pukyong National University Busan 48513 Republic of Korea

**Keywords:** electrical conductivity, mechanical modulus, polyacrylonitrile‐based carbon film, single‐walled carbon nanotube, templating–coalescing behavior

## Abstract

Promoting the feasibility of carbon films as electrode applications requires sufficient performances in view of both electrical and mechanical properties. Herein, carbon films with ultrahigh electrical conductivity and mechanical modulus are prepared by high temperature carbonization of polyacrylonitrile (PAN)/single‐walled carbon nanotube (SWNT) nanocomposites. Achieving both performances is ascribed to remarkable graphitic crystallinity, resulting from the sequential templating–coalescing behavior of concentrated SWNT bundles (B‐CNTs). While well‐dispersed SWNTs (WD‐CNTs) facilitate radial templating according to their tubular geometry, flattened B‐CNTs sandwiched between carbonized PAN matrices induce vertical templating, where the former and latter produce concentric and planar crystallizations of the graphitic structure, respectively. After carbonization at 2500 °C with the remaining WD‐CNTs as microstructural defects, the flattened B‐CNTs coalesce into graphitic crystals by zipping the surrounding matrix, resulting in high crystallinity with the crystal thicknesses of 27.4 and 39.4 nm for the (002) and (10) planes, respectively. For comparison, the graphene oxide (GO) containing carbon films produce a less‐ordered graphitic phase owing to irregular templating, despite the geometrical consistency. Consequently, PAN/B‐CNT carbon films exhibit exceptional electrical conductivity (40.7 × 10^4^ S m^−1^) and mechanical modulus (38.2 ± 6.4 GPa). Thus, controlling the templating−coalescing behavior of SWNTs is the key for improving final performances of carbon films.

## Graphical abstract

Carbon films with exceptional electrical conductivity and mechanical modulus are prepared using polyacrylonitrile/single‐walled carbon nanotube (SWNT) nanocomposites through carbonization (2500 °C). High graphitic crystallinity is ascribed to the sequential templating−coalescing behavior of SWNT bundles. Contrary to the well‐dispersed SWNTs, the flattened SWNT bundles induce vertical templating and planar graphitic crystallization. Further, few SWNT bundles remain as defects owing to coalescence.

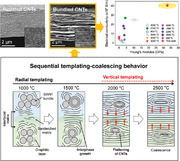

## 1 Introduction

Owing to their outstanding electrical functionality and thermal stabilities, carbon films have garnered great interest as electrode materials in the fields of energy storage and electronic devices.^[^ ^1^, ^2^, ^3^, ^4^ ^]^ Carbon films can be formed by an assembly of carbon nanomaterials. However, recent studies have focused on the use of polymeric precursors, including polyacrylonitrile (PAN) and polyimide, because of the high processability and easy fabrication of large‐sized films.^[^ ^5^, ^6^, ^7^ ^]^ However, the less‐organized polymeric matrix with mixed phase of amorphous and crystalline regions is converted to a turbostratic structure during carbonization, which results in insufficient performances of carbon films as electrode applications from the perspective of both electrical and mechanical properties.^[^ ^8^, ^9^, ^10^ ^]^ The graphitic crystallinity of carbon films and their final performance can be enhanced by incorporating different types of carbon nanofillers, including graphene oxide (GO) and single‐walled carbon nanotube (SWNT) as the graphitic template.^[^ ^11^, ^12^, ^13^, ^14^, ^15^ ^]^

In nanocomposites, the interfacial interaction of a polymer and nanofiller dramatically improves the mechanical properties owing to the heterogeneous crystal nucleation of the matrix and intrinsically superior properties of the filler.^[^ ^16^, ^17^, ^18^ ^]^ In addition, the preferential arrangement of the crystallites on the filler surface obtained by the templating effect greatly improves the microstructure orientation.^[^ ^19^, ^20^, ^21^, ^22^ ^]^ This templating effect is referred to as the pseudoepitaxial crystal growth, and the microstructural region composed of the templated crystals is known as the interphase.^[^ ^5^, ^23^ ^]^ Hence, the both electrical and mechanical performances of carbon films from polymeric nanocomposites are dominantly determined by the extent and microstructural alignment of the interphase.

In the preparation of carbon films from nanocomposite precursors, most researchers have followed two empirical conventions. First, a nanofiller used for the nanocomposites is expected to have the same geometry as that of the desired composite.^[^ ^24^ ^]^ While SWNT (tubular geometry) is preferred for carbon fibers, GO (planar geometry) is preferred for carbon films because the macroscopic alignment of the graphitic sheets to the major axis of the products may increase the performance in the corresponding direction. That is, the structural evolution prefers to the geometrical consistency of the components. In this regard, the geometry of the nanofillers is a major factor for the order of the crystalline interphase, and the macroscopic order and morphology of the final carbon materials.^[^ ^25^ ^]^ Second, the higher dispersion state of nanofillers increases the reinforcing (or templating) efficiency in the nanocomposites.^[^ ^15^, ^26^ ^]^ A well‐dispersed state is an indispensable condition for facilitating the nanoeffect (i.e., extremely high surface area). Although GO exhibits improved dispersibility and miscibility with polymers, the poor dispersibility of SWNT may severely lower its efficiency to be less than the theoretical expectations.^[^ ^27^, ^28^ ^]^ According to these two conventions, numerous studies on the preparation of nanocomposite carbon films have adopted GO as the templating agent for improving the graphitic crystallinity instead of SWNTs.^[^ ^12^, ^24^, ^29^ ^]^

**Figure 1:**
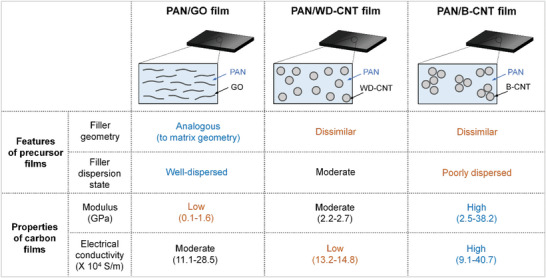
Comparison of features of three different nanocomposite precursor films and final properties of their resulting carbon films.

In this study, we prepared PAN‐based nanocomposite carbon films with ultrahigh electrical conductivity (up to 40.7 × 10^4^ S m^−1^) and mechanical modulus (up to 38.2 GPa) by incorporating SWNT bundle (B‐CNT), which overturned the aforementioned issues as depicted in **Figure** **1**. Given the aforementioned empirical conventions, B‐CNT seems to be unsuitable option as a reinforcing filler rather than GO and well‐dispersed SWNT (WD‐CNT) owing to the geometrical inconsistency and poor dispersion state in PAN precursor film. In terms of both electrical and mechanical performances, however, final properties of PAN/B‐CNT carbon films are superior to those of the other nanocomposite carbon films (Figure 1). From the perspective of the filler geometry, the higher templating efficiency of B‐CNT than that of GO was obtained during the carbonization of PAN films, despite the geometrical mismatch between the tubular nanofiller and planar film matrix. This suggests that nanofiller geometry is no longer a priority in the preparation of high‐quality graphitic films from nanocomposites. From the perspective of the filler dispersion, the B‐CNT offered a higher templating efficiency and crystallinity of the carbonized PAN nanocomposite films than that of the WD‐CNT at an identical filler loading. To determine the reasons for these phenomena, PAN‐based carbon films with B‐CNTs were prepared at a filler loading of 1–15 wt% through carbonization and graphitization up to 2500 °C. In addition, the templating and coalescing behaviors of carbon nanofillers were investigated based on the macroscopic and microscopic observations. For comparison, WD‐CNT and GO‐containing carbon films were also studied.

## 2 Results and Discussions

### 2.1 Templating and Coalescing Behaviors of SWNTs in Carbon Films

Graphitic film can be conveniently manufactured by the carbonization and graphitization of PAN precursor films; however, its microstructure mainly consists of a turbostratic structure, which is far from the ideal graphitic structure.^[^ ^30^, ^31^ ^]^ To improve the crystallinity through the conversion of the turbostratic to graphitic structures, SWNTs were incorporated into PAN films, and their templating effect on the graphitic crystal growth of the polymeric matrix was investigated with respect to the filler dispersion state and amount. Two types of PAN/SWNT nanocomposite precursor films were prepared as shown in Figure S1 (Supporting Information). For the WD‐CNTs, nanocomposites were prepared by the repeated addition of diluted SWNT dispersion (5 mg L^−1^ in *N,N′*‐dimethylformamide (DMF)) and vacuum distillation. The nanocomposites with B‐CNTs were prepared by adding a concentrated SWNT paste (7.2 g L^−1^ in DMF). Figure S2 (Supporting Information) shows the cross‐sectional scanning electron microscopy (SEM) images of precursor nanocomposite films with 1 wt% B‐CNT and WD‐CNT. The two nanocomposite systems exhibited huge difference in the dispersion state depending on the preparation routes. It is an undeniable fact the SWNT/DMF paste, which has a concentration of SWNT 1440 times greater than the diluted SWNT/DMF dispersion, develops a worse dispersion state. Hereafter, the carbonized films are labelled as XY‐Z, where X, Y, and Z are the kind of nanofiller, nanofiller concentration with respect to the polymer, and carbonization temperature, respectively. For example, PAN film with 7 wt% WD‐CNTs carbonized at 2500 °C is denoted as WD‐CNT7‐2500.

**Figure 2:**
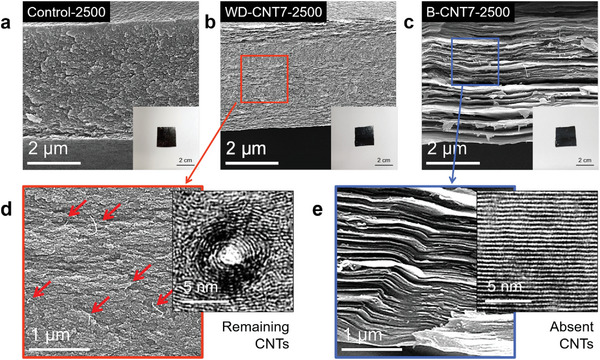
The macroscopic and microscopic morphologies of polyacrylonitrile (PAN)‐based carbonized films depending on the dispersion state of single‐walled carbon nanotubes (SWNTs). Scanning electron microscopy (SEM) images of a) control‐2500, b) WD‐CNT7‐2500, and c) B‐CNT7‐2500 films. Photographs of the corresponding carbon films are also shown as inset figures. The magnified SEM and high‐resolution transmission electron microscopy (HR‐TEM) images of d) WD‐CNT7‐2500 and e) B‐CNT7‐2500 films, respectively.

Depending on the dispersion state of the SWNTs, the nanocomposite films exhibited contrasting morphologies after carbonization at 2500 °C (**Figure** **2**a–c). As shown in the cross‐sectional SEM images, the WD‐CNT7‐2500 film exhibited an irregular morphology with a small fraction of aligned structure (Figure 2b), whereas the B‐CNT7‐2500 film possessed a distinct sheet‐like structure (Figure 2c). This morphological difference reveals the stronger templating effect of the B‐CNTs than that of the W‐CNTs at the same filler loading. In addition, SWNTs were rarely observed in the B‐CNT film after carbonization, in contrast to the remaining SWNTs in the WD‐CNT film (Figure 2d,e). Owing to the geometrical discrepancy between the matrix and filler, the remaining SWNT molecules may act as structural defects in the carbon film because the concentric crystal structure templated on the nanotube wall severely degrades the structural homogeneity and crystallite orientation (high‐resolution transmission electron microscopy (HR‐TEM) image in Figure 2d).

**Figure 3:**
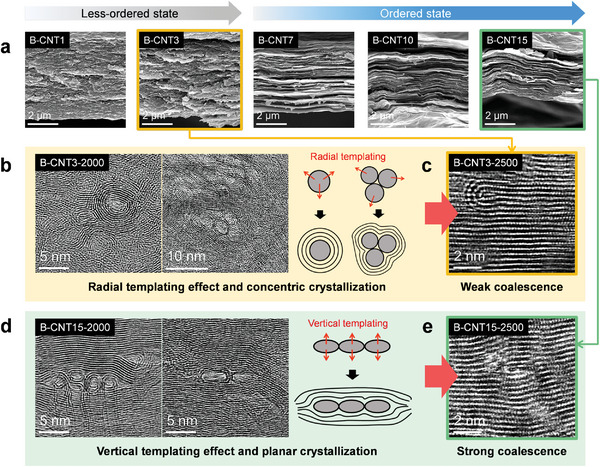
Structural evolution mechanisms of polyacrylonitrile (PAN)/single‐walled carbon nanotube bundle (B‐CNT) carbon films at different filler loadings. a) Scanning electron microscopy (SEM) images of films carbonized at 2500 °C containing different B‐CNT contents. Transmission electron microscopy (TEM) images and schematic descriptions of b) radial (B‐CNT3) and d) vertical templating effects (B‐CNT15) and c,e) resulting coalescing behaviors, respectively.

The strong templating effect of the B‐CNTs can be explored by observing the microstructural evolution of the B‐CNT‐2500 films with 1–15 wt% nanofiller (**Figure** **3**). The carbonized films with 1 and 3 wt% B‐CNTs exhibited disordered morphologies, whereas a sheet‐like graphitic structure appeared with a B‐CNT concentration of more than 7 wt%, which is ascribed to the different templating mechanisms and efficiencies of the B‐CNTs at different filler loadings (Figure 3a). The different templating at low and high B‐CNT loadings are compared in Figure 3b–e, indicating the graphitic crystallization growth of the B‐CNT3 and B‐CNT15 films at a carbonization temperature of 2000 °C, respectively. For the B‐CNT3‐2000 film, individual SWNT molecules and small bundles were dispersed and isolated (Figure S3a, Supporting Information), which primarily induced the radial templating effect on the graphitization of the neighboring PAN matrix (Figure 3b). Radial templating eventually resulted in the concentric crystallization of the graphitic structure.^[^ ^32^, ^33^ ^]^ With further carbonization at 2500 °C, the stiffness of the SWNT molecules surrounded by a concentrically templated structure increased, thereby inhibiting the coalescence into the graphitized PAN film matrix (Figure 3c). Hence, SWNTs and templated structures remained at 2500 °C.

At a high filler loading of more than 7 wt%, the B‐CNTs overlap, and their interaction (i.e., crowding by entanglement) created a sandwiched matrix region, thereby expanding the interphase (Figure S3b, Supporting Information). As a result, the B‐CNTs were tightly confined between the aligned interphases, followed by the flattening of the bundles (Figure 3d). The flattening reveals that the surrounding interphases strongly pressurize the sandwiched B‐CNTs. As the carbonization proceeds at the elevated temperatures, the interphases become thicker and larger, resulting in the zipping of the surrounding interphases and subsequent collapse of the tubular structure. Therefore, the confinement pressure, responsible for the flattening of B‐CNTs, indicates the pressure applied to the sandwiched bundles between the growing interphases. The flattened B‐CNTs promoted a vertical templating effect on the neighboring graphitic crystals, as opposed to the inherent 1D nanotubes. Consequently, vertical templating facilitated the planar crystallization of the interphase, which promoted the structural alignment of the carbon films because of the axial consistency of the planar microstructure and macroscopic carbon film. Moreover, the flattened B‐CNTs experienced strong coalescence upon further carbonization at 2500 °C (Figure 3e). Thus, the sequential process of the vertical templating–coalescing behavior of the B‐CNTs afforded a remarkably high crystallinity of the graphitic structure with minimal structural defects.

**Figure 4:**
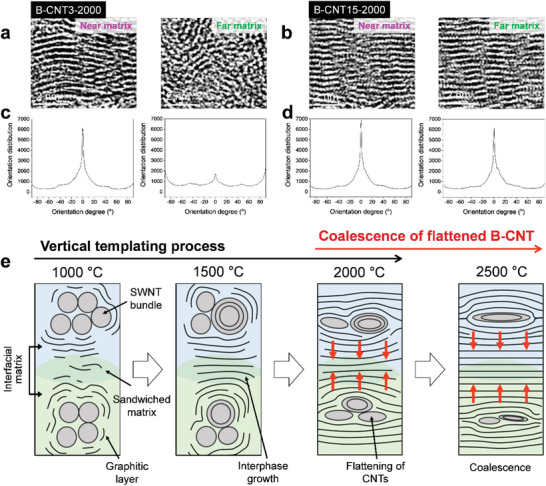
Different templating efficiencies of single‐walled carbon nanotube bundles (B‐CNTs) depending on the filler loadings. High‐resolution transmission electron microscopy (HR‐TEM) images of a) B‐CNT3‐2000 and b) B‐CNT15‐2000. Orientation distribution along the in‐plane film axis (horizontal direction of images) of c) B‐CNT3‐2000 and d) B‐CNT15‐2000. e) A schematic presentation of the sequential templating‐coalescing behavior of B‐CNTs during carbonation of the films above the critical filler loading.

The different templating mechanisms with 3 and 15 wt% B‐CNTs affected the templating efficiency. Thus, we evaluated the degree of alignment of the graphitic structures near (<10 nm from the bundle template) and far (≈20 nm from the template) from the matrices (**Figure** **4**). For B‐CNT3‐2000, which has dominant radial templating effect and a highly ordered graphitic structure in the proximate matrix, the far region exhibited irregular alignment (Figure 4a). In contrast, in B‐CNT15‐2000, which has preferable vertical templating effect, the ordering of the graphitic structure in the far region is as high as that in the nearby region (Figure 4b). This indicates the far‐reaching effect of vertical templating, unlike radial templating. As a result, far‐reaching templating can further expand the highly ordered interphase (Figure S3b, Supporting Information). The image analysis of the degree of anisotropic orientation can provide clear evidence to assess the ordering of the graphitic layers.^[^ ^34^, ^35^ ^]^ In B‐CNT3‐2000, the region near the matrix exhibited a strong and narrow peak in the radial summation plot of the pixel intensities, indicating a higher anisotropic alignment, whereas the region far from the matrix exhibited a scattered plot, suggesting a low templating efficiency (Figure 4c). However, the sharp peaks in the radial plots for both regions in B‐CNT15‐2000 indicate a high templating efficiency (Figure 4d). The sequential templating–coalescing behavior of B‐CNTs above the critical filler loading is schematically summarized in Figure 4e. At the B‐CNT concentrations of more than 7 wt%, the vertical templating process took place preferentially (1000−2000 °C), followed by the coalescence of the flattened B‐CNTs (2000−2500 °C). In the early stage, the crowding of B‐CNTs produces the sandwiched interfacial matrix, which is converted to the aligned interphase. The expansion of the aligned interphase confined and pressurized the bundles, inducing the flattening of B‐CNTs (Figure 3d). As a result, the flattened bundles promote the vertical templating and then ultimately coalesced into the matrix.

**Figure 5:**
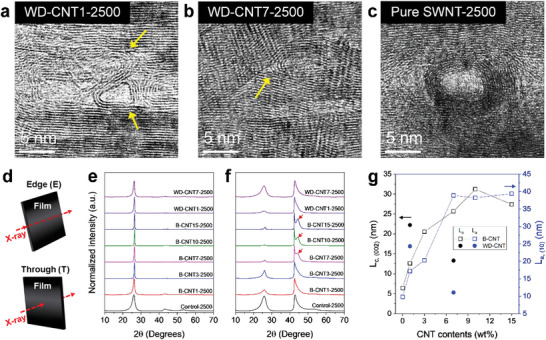
Transmission electron microscopy (TEM) images of a) WD‐CNT1‐2500, b) WD‐CNT7‐2500, and c) pure single‐walled carbon nanotube (SWNT) films carbonized at 2500 °C. The yellow arrows indicate the remaining well‐dispersed single‐walled carbon nanotubes (WD‐CNTs) without coalescence. d) A scheme describing the direction in which the wide‐angle X‐ray diffraction (WAXD) data of films are measured. WAXD profiles of 2500 °C‐carbonized films measured in e) E‐ and f) T‐directions, respectively. g) The crystal thickness (*L*_c_) and in‐plane crystal size (*L*_a_) calculated from (002) and (10) peaks, respectively.

The remaining SWNT molecules were easily observed in the WD‐CNT carbon films (Figure 2d), indicating a weak coalescence even at a carbonization temperature of 2500 °C. **Figure** **5**a–c shows the microstructures of the WD‐CNT and pure SWNT carbon films carbonized at 2500 °C. Although the WD‐CNTs were distorted and slightly collapsed, they were retained with filler contents of 1 and 7 wt% (Figure 5a,b). For the pure SWNT film, the nanotube structure was more distinct (Figure 5c). The evolution of the multiwalled CNTs by coalescence or concentric crystallization from the SWNTs stiffened the nanotubes, which stabilized their tubular structure.^[^ ^36^, ^37^, ^38^, ^39^ ^]^ The crystallinity and alignment of the templated graphitic structure of the carbonized films were evaluated by analyzing the wide‐angle X‐ray diffraction (WAXD) results in the edge (E) and through (T) directions of the films (Figure 5d). For an ideal graphite film with the graphitic crystallites oriented along the in‐plane film direction, the E‐direction pattern (E‐spectrum) exclusively shows the (00*l*) plane, whereas the T‐direction is expected to show the (10) plane.^[^ ^40^, ^41^ ^]^ In the control‐2500 film, a broad (002) peak at approximately 2*θ* of 26° was noted in the E‐spectrum (Figure 5e), which was also observed in the T‐spectrum owing to the poor alignment along the in‐plane direction of the film (Figure 5f). However, in the nanocomposite system, the E‐ and T‐spectral patterns followed ideal trends with an overwhelmingly strong (002) peak in the E direction (Figure 5e) and (10) peak in the T‐direction (Figure 5f), which verified the improved crystal alignment. Particularly, in B‐CNT‐2500 at a filler loading of more than 7 wt%, the carbonized films produced sharp diffraction peaks in both directions owing to the well‐ordered graphitic structure. In addition, the out‐of‐plane (L_c_) and in‐plane (L_a_) crystal thicknesses were calculated from the (002) in the E‐spectra and (10) in the T‐spectra, respectively. L_c_ and L_a_ were significantly larger at the B‐CNT content of more than 7 wt% than those with low B‐CNT contents (Figure 5g), which confirm the high crystallinity of the templated graphitic structure in the B‐CNT‐2500 films above the critical filler content, as observed by TEM (Figure 3d,e). The highly ordered graphitic structure of the B‐CNT films was confirmed by the appearance of the (101) peak at 2*θ* of approximately 45° in the T‐spectra, which can only be observed when a high degree of out‐of‐plane stacking and in‐plane ordering concurrently exists (red arrows in Figure 5f).^[^ ^15^, ^42^, ^43^ ^]^

### 2.2 Comparison of the Templating Efficiency of SWNTs and GO

**Figure 6:**
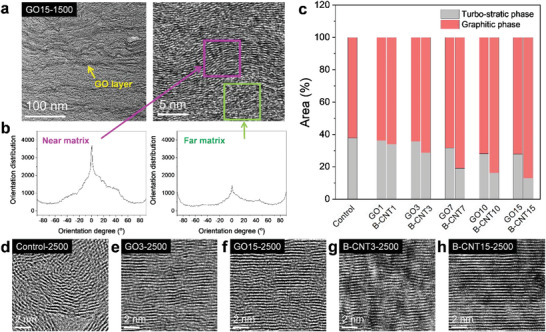
a) Transmission electron microscopy (TEM) images and b) orientation distribution of GO15‐1500 films. c) The fraction of turbostratic and graphitic phases of 2500 °C‐carbonized films, calculated by deconvolution of (002) peak from wide‐angle X‐ray diffraction (WAXD) profiles. High‐resolution transmission electron microscopy (HR‐TEM) images showing difference in microstructure and its orientation along the in‐plane axis of d) control‐2500, e) GO3‐2500, f) GO15‐2500, g) B‐CNT3‐2500, and h) B‐CNT15‐2500.

Conventionally, the sheet‐like geometry of GO is preferable for preparing nanocomposite carbon films owing to its geometrical consistency.^[^ ^24^ ^]^ As observed in the HR‐TEM images of the GO15‐1500 film, the high flexibility of the thin graphene sheet resulted in severe crumpling and wrinkling in the carbonized matrix (**Figure** **6**a). Analogous to the WD‐CNT systems, the GO template improved the alignment near the interfacial matrix, whereas its templating effect on the far matrix was insufficient, as confirmed by the analysis of the degree of anisotropic alignment of the graphitic layers (Figure 6b). According to its planar geometry, this suggests a low templating efficiency despite the vertical templating effect. The low efficiency of GO is ascribed to the low surface rigidity of the flexible graphene sheets. According to a simulation of Saha et al.,^[^ ^13^ ^]^ the fluctuating and crumpled behavior of GO reduces the catalytic activity in the templating process during carbonization. In contrast, the rigid surface of CNTs is favorable to physisorption and chemisorption, thereby increasing the templating efficiency. Thus, the molecular flexibility and surface rigidity of carbon fillers are primary factors in determining the templating efficiency.

The different templating efficiencies of WD‐CNTs and B‐CNTs can be elucidated in the same vein. Liew et al.^[^ ^44^ ^]^ reported that the bundle assembly of individual SWNTs can achieve a fivefold increase in the buckling load, indicating a significantly higher rigidity of the bundled state than the individual molecules. As a result, a more durable template based on B‐CNTs can act as a stronger carbon foundation. Comparing the graphitic crystallinity of the B‐CNT‐2500 and GO‐2500 systems, the fraction of the graphitic to turbostratic structures (G/T ratio) from the deconvolution of the (002) peak was significantly higher in the B‐CNT films than in the GO films (Figure 6c).^[^ ^45^, ^46^ ^]^ In particular, at 15 wt%, the G/T ratio of the B‐CNT15 film (6.73) was 2.6 times higher than that of the GO15 film (2.59). Aforementioned, the templating process is referred to as the conversion from the turbostratic to graphitic structures through the pseudoepitaxial growth. In this regard, the G/T ratio is suitable quantitative parameter for defining the templating efficiency of the carbon nanofiller in each nanocomposite film. The highest graphitic crystallinity and relevant templating efficiency of the B‐CNT15‐2500 film among the carbonized films were confirmed by the HR‐TEM images (Figure 6d–h).

**Figure 7:**
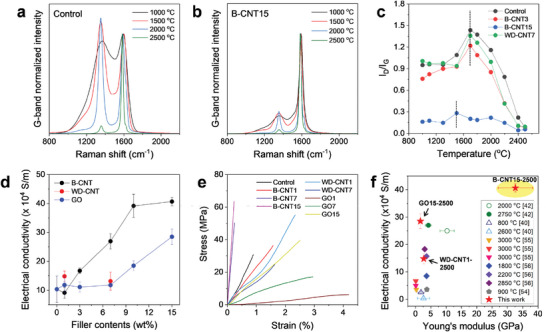
Raman spectra and various properties of carbon films. Raman spectra of a) control and b) B‐CNT15 films, and c) variation of *I*_D_/*I*_G_ ratio of various carbon films with respect to the carbonization temperature. d) The electrical conductivities and e) stress–strain curves of films carbonized at 2500 °C. f) Ashby plot for comparing electrical conductivities and Young's moduli of various carbon films.

According to previous studies,^[^ ^14^, ^15^ ^]^ the filler‐induced templating effect accelerates the structural change of the PAN matrix during heat treatment, which enables low‐temperature carbonization. The microstructural evolution of the control and nanocomposite films was traced by analyzing the Raman spectra of the carbonized films over a temperature range of 1000–2500 °C (**Figure** **7**a,b and Figure S4, Supporting Information). The Raman spectra were deconvoluted based on the D‐band at approximately 1350 cm^−1^ (disordered graphitic structure or defect) and G‐band at approximately 1580 cm^−1^ (ordered graphitic structure).^[^ ^47^, ^48^, ^49^, ^50^, ^51^ ^]^ As shown in the experimental spectra (Figure 7a,b and Figure S4, Supporting Information), the carbonized nanocomposite films exhibited a lower intensity of the D‐band than that in the control films over the entire temperature range because of the templating effect and/or the presence of the carbonaceous fillers.^[^ ^52^, ^53^ ^]^

To trace the structural changes with increasing carbonization temperature, the variations in the intensity ratio of the D‐ to G‐bands (*I*_D_/*I*_G_) from 1000 °C to 2500 °C are plotted in Figure 7c. As the carbonization temperature increases, the *I*_D_/*I*_G_ values of the control and nanocomposite systems varied through three distinct stages, as reported in our previous research:^[^ ^47^ ^]^ (1) the formation of the turbostratic graphitic structure (insignificant change in *I*_D_/*I*_G_), (2) the phase conversion to a polycrystalline graphitic structure (sharp increase in *I*_D_/*I*_G_), and (3) the in‐plane homogenization of highly ordered graphitic structure (sharp reduction in *I*_D_/*I*_G_). The evolution of the highly crystalline graphitic structure occurred between the 2^nd^ and 3^rd^ stages. As shown in Figure 7c, the control films underwent this transition at 1700 °C. For the WD‐CNT‐ and B‐CNT‐containing films, the transition temperatures of the WD‐CNT7 and B‐CNT3 systems are analogous to those of the control film, whereas those of B‐CNT15 shifted to 1500 °C. The shift to a lower temperature for B‐CNT15 suggests the accelerated carbonization compared to the control and other composite systems, which agrees well with the high templating efficiency. However, owing to its low templating efficiency, the GO‐containing nanocomposite films exhibited a transition temperature of 1700 °C, analogous to that of the control film (Figure S5, Supporting Information).

The electrical and mechanical performance of the carbon films reflect their microstructural integrity (Figure 7d–f). Owing to the high crystallinity and well‐aligned graphitic structure of the B‐CNT graphitic films, they exhibited superior electrical conductivity after carbonization at 2500 °C (Figure 7d and Table S1, Supporting Information). At 15 wt%, the conductivity of the B‐CNT‐2500 film (40.7 × 10^4^ S m^−1^) was 1.4 times higher than that of the GO‐2500 film (28.5 × 10^4^ S m^−1^). In terms of the mechanical properties, the high crystallinity of the B‐CNT films at filler loadings of more than 7 wt% results in an extremely high Young's moduli (Figure 7e and Table S1, Supporting Information). The modulus of B‐CNT7‐2500 (29.1 ± 2.1 GPa) is 11.6 times higher than that of B‐CNT7‐2500 (2.5 ± 0.2 GPa). In comparison with GO, moreover, the modulus of B‐CNT15‐2500 (32.6 ± 5.6 GPa) is 20.4 times higher than that of GO15‐2500 (1.6 ± 0.7 GPa). As shown in the Ashby plot of the electrical conductivity versus Young's modulus for comparison with other reported graphitic films (Figure 7f),^[^ ^40^, ^42^, ^54^, ^55^, ^56^ ^]^ the B‐CNT15‐2500 films in the current study exhibited the highest electrical and mechanical performance.

## 3 Conclusion

PAN/SWNT‐based nanocomposite carbon films with ultrahigh electrical conductivity and mechanical modulus as well as remarkable graphitic crystallinity were fabricated by carbonization at 2500 °C. In addition, the microstructural evolution of the PAN‐based carbon films during carbonization was investigated in terms of the filler geometry, amount, and dispersion state. The crystallinity of the carbon films was predominantly dependent on the templating efficiency and coalescence behavior of the carbon nanofillers. For SWNT‐containing nanocomposites, the WD‐CNT induced concentric crystallization of the graphitic structure by the radial templating effect according to the inherent tubular structure, whereas the flattened B‐CNTs caused planar crystallization by vertical templating. In addition, the templating effect of the B‐CNTs reached approximately 20 nm, significantly expanding the highly aligned interphase. Consequently, the B‐CNTs coalesced into a highly crystalline graphitic structure by zipping the surrounding interphase. In comparison, GO exhibited a low templating efficiency and crystallinity of the carbon films, despite the geometrical consistency, because the crumpled graphene sheets with low surface rigidity resulted in an irregular templating growth. We believe that our results establish a foundation for the relationship between the nanofiller parameter and evolution of graphitic structures.

## 4 Experimental Section

### Materials

PAN with an average molecular weight of approximately 250 000 g mol^−1^ was obtained from Japan Exlan Co. Ltd. (Japan) and used after vacuum drying at 80 °C for 24 h. GO and SWNT powders were purchased from Graphenea Co. (USA) and OCSiAl Co. (Luxembourg), respectively. Figure S6 (Supporting Information) shows the TEM images of GO and SWNT used in the current study. According to the data sheet from manufacturers, while GO has the lateral size and thickness of 0.58 ± 0.40 µm and ≈1 nm, respectively, SWNT has the diameter and length of 1.6 ± 0.4 nm and >5 µm, respectively. According to the Raman spectra (Figure S7, Supporting Information), *I*_D_/*I*_G_ ratios of GO and SWNT were 1.07 and 0.02, respectively. Reagent‐grade DMF was supplied by Samchun Co. Ltd. (Korea).

### Dispersion of the Nanofillers and Solution Preparation

PAN powder was dissolved in DMF at a concentration of 7 g/100 mL to prepare the control solution using an overhead mixer at 70 °C for 6 h. Nanocomposite solutions were prepared as follows. For the GO dispersion, GO powder was dispersed in 4 mg mL^−1^ DMF by stirring and bath sonication for 5 h to obtain an optically homogeneous solution. In addition, for WD‐CNT, SWNT powders were dispersed in 5 mg L^−1^ DMF by homogenization for 30 min (T18 digital, IKA, Germany), followed by bath sonication for 24 h (CPX5800, Branson, USA). Compared to GO/DMF, the lower dispersion concentration of SWNT/DMF is ascribed to the difficulty of dispersion by strong van der Waals interactions between the individual SWNTs, whereas the GO become readily dispersed because of their functional groups on the surface. Well‐dispersed GO or SWNT dispersions were obtained by mixing with the separately prepared PAN solutions, and the excess solvent was removed by vacuum distillation while stirring to obtain the same solid concentration as the control PAN solution. To observe the effects of the degree of SWNT dispersion on the templating effect, B‐CNTs were obtained by directly mixing the PAN solution with a highly concentrated SWNT paste. The SWNT paste (7.2 g L^−1^) was prepared by centrifugation at 10 000 rpm and bath sonication. The B‐CNT nanofiller concentrations with respect to the polymer were 1, 3, 7, 10, and 15 wt%, and the WD‐CNT‐based nanocomposite films were prepared with the CNT concentrations of 1 and 7 wt% only.

### Preparation of the PAN Precursor Films and Heat Treatment

The prepared solutions were cast into a film on a glass substrate using a doctor blade, followed by drying at 40 °C in a convection oven for 1 d. For the pure SWNT film, the well‐dispersed CNT/DMF solution (5 mg L^−1^) was vacuum‐filtered and then dried at 40 °C. Heat treatments, including stabilization and carbonization, were conducted by placing the film specimens between two graphite plates. Both plates were fixed after adjusting the thickness of the precursor films. To apply the same stress to all the specimens during heat treatment, the films were cut to the same size of 80 × 25 mm. The precursor films were stabilized at 260 °C for 5 h at a heating rate of 3 °C min^−1^ in air using a box furnace, and then naturally cooled. Subsequently, the films were carbonized in the temperature range 1000–2500 °C in steps of 100 °C at a heating rate of 5 °C min^−1^ in an inert atmosphere.

### Characterization

The cross‐sectional morphologies of the carbonized films were observed using field‐emission SEM (Nanonova 230, FEI Co., USA) at an accelerating voltage of 10 kV after sputter‐coating with Pt at 20 mA for 30 s. The microstructure of the carbonized samples was observed using HR‐TEM (JEM‐2100, JEOL, Japan) operated at an accelerating voltage of 200 kV. Crystallographic information was evaluated using a synchrotron source at the PLS‐II 6D UNIST‐PAL beamline of the Pohang Accelerator Laboratory. The energy of the X‐rays was 18.986 keV (*λ* = 0.653 Å). The WAXD patterns of the carbonized and graphitized films were obtained in the T and E directions, respectively. Raman spectra were collected from five different sites with an exposure of 1 s and accumulation of 150 times for each sample using an Alpha 300s micro‐Raman spectrometer (WITec, Germany). The excitation wavelength and laser power were 532 nm and 0.5 mV, respectively. For the analysis, the WAXD and Raman spectra were deconvoluted using the Peakfit software package (Seasolve, USA). The electrical conductivity was measured by the four‐probe method (CMT‐SR2000N, Advanced Instrument Technology, USA) at five different sites for each sample. For the four‐point probe test, a fixed current was passed through the two outer probes and a voltage was measured between two inner probes. The conductivity (S m^−1^) of the film was calculated from the reciprocal value of the resistivity (conductivity (S m^−1^) = 1/resistivity (Ω m)), and the resistivity was obtained by multiplying the sheet resistance (Ω square^−1^) and thickness of the film (resistivity (Ω m) = sheet resistance (Ω square^−1^) × thickness (m)). The film thickness was determined by their cross‐sectional SEM images and Image J software. The mechanical properties of films were measured by dynamic mechanical analysis (DMA, Q800, TA Instrument Inc., USA) at a gauge length of 1 inch. As an input parameter, the cross‐sectional area of each film was calculated by multiplying its width and thickness (it was assumed that the cross‐sectional shape of films was rectangular). All the measured films were cut uniformly with a width of 5 mm, and the thickness of each specimen was determined by SEM images. The slope in the linear range (mostly, 0.2−0.4%) of the stress–strain curve was calculated for obtaining Young's modulus. The anisotropic alignment of the graphitic structure observed by TEM was determined using two‐dimensional fast Fourier transform analysis and radial summation using Image J.

## Conflict of Interest

The authors declare no conflict of interest.

## Supporting information

Supporting Information

## Data Availability

The data that support the findings of this study are available from the corresponding author upon reasonable request.
