# Supplementary material for: Achieving Both Ultrahigh Electrical Conductivity and Mechanical Modulus of Carbon Films: Templating‐Coalescing Behavior of Single‐Walled Carbon Nanotube in Polyacrylonitrile
Source: Adv Sci (Weinh). 2023 Jan 22;10(8):2205924. doi: 10.1002/advs.202205924 (PMC10015862; doi:10.1002/advs.202205924)
Supplement: Supplementary file 1 — Supporting Information [file ADVS-10-2205924-s001.pdf]

ADVANCED SCIENCE
Open Access

Supporting Information

for *Adv. Sci.*, DOI 10.1002/advs.202205924

Achieving Both Ultrahigh Electrical Conductivity and Mechanical Modulus of Carbon Films: Templating-Coalescing Behavior of Single-Walled Carbon Nanotube in Polyacrylonitrile

Jung-Eun Lee, Jung Hoon Kim, Joong Tark Han, Han Gi Chae and Youngho Eom**

Supporting Information

Achieving both ultrahigh electrical conductivity and mechanical modulus of carbon films: Templating−coalescing behavior of single-walled carbon nanotube in polyacrylonitrile

Jung-Eun Lee[1], *Jung Hoon Kim*[2], *Joong Tark Han*[2, 3], *Han Gi Chae*[1,*], *and Youngho Eom*[4,*]

[1]Department of Materials Science and Engineering, Ulsan National Institute of Science and Technology (UNIST), Ulsan 44919, Republic of Korea

[2]Nano Hybrid Technology Research Center, Korea Electrotechnology Research Institute (KERI), Changwon-si, Gyeongsangnam-do, 51543, Republic of Korea

[3]Electrical Functional Material Engineering, Korea University of Science and Technology (UST), Changwon-si, Gyeongsangnam-do, 51543, Republic of Korea

[4]Department of Polymer Engineering, Pukyong National University, Busan, 48513, Republic of Korea

E-mail: hgchae@unist.ac.kr (H. G. Chae), eomyh@pknu.ac.kr (Y. Eom)

Table S1. Mechanical and electrical properties of various films carbonized at 2500 °C

Nanofiller	Filler contents (wt.%)	Mechanical strength (MPa)	Young's modulus (GPa)	Electrical conductivity (x10^4 S/m)
Control	0	35.3 ± 6.4	3.0 ± 0.6	10.4 ± 4.6
GO	1	6.4 ± 2.7	0.1 ± 0.0	11.8 ± 3.1
	3	10.2 ± 1.9	0.7 ± 0.4	11.1 ± 1.8
	7	18.7 ± 2.1	1.5 ± 0.2	11.8 ± 0.3
	10	26.0 ± 5.8	1.6 ± 0.4	18.5 ± 1.7
	15	32.3 ± 13.2	1.6 ± 0.7	28.5 ± 2.7
B-CNT	1	36.3 ± 2.1	2.5 ± 0.2	9.1 ± 1.9
	3	32.1 ± 1.8	2.5 ± 0.2	16.8 ± 0.9
	7	51.2 ± 10.0	29.1 ± 2.1	27.0 ± 2.7
	10	52.0 ± 12.8	38.2 ± 6.4	39.2 ± 4.0
	15	63.4 ± 15.1	32.6 ± 5.6	40.7 ± 1.5
WD-CNT	1	45.0 ± 7.3	2.7 ± 0.5	14.8 ± 1.9
	7	26.7 ± 5.5	2.2 ± 0.7	13.2 ± 3.1

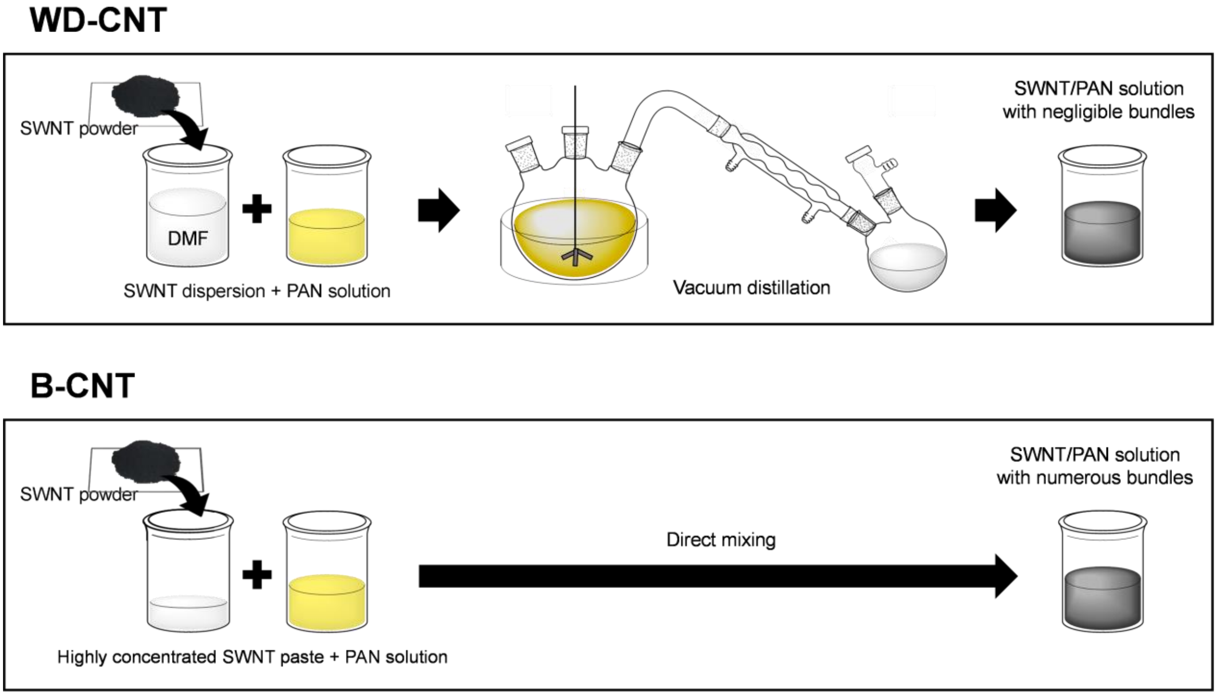

Figure S1. Schematic of detailed preparation procedure of WD-CNT- or B-CNT-containing PAN-based nanocomposite solutions.

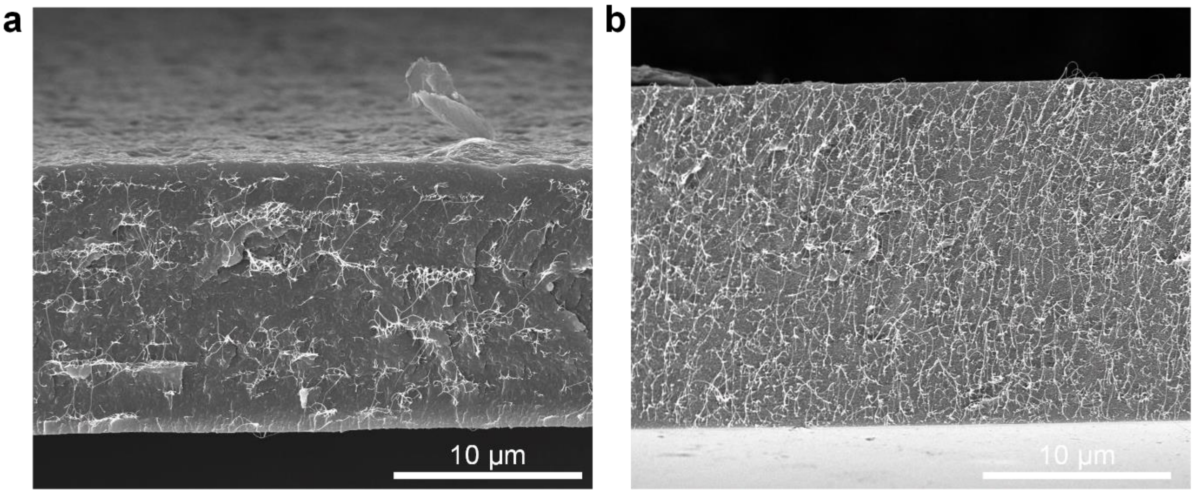

Figure S2. The cross-sectional SEM images of precursor PAN nanocomposite films with 1 wt.% of (a) B-CNT and (b) WD-CNT.

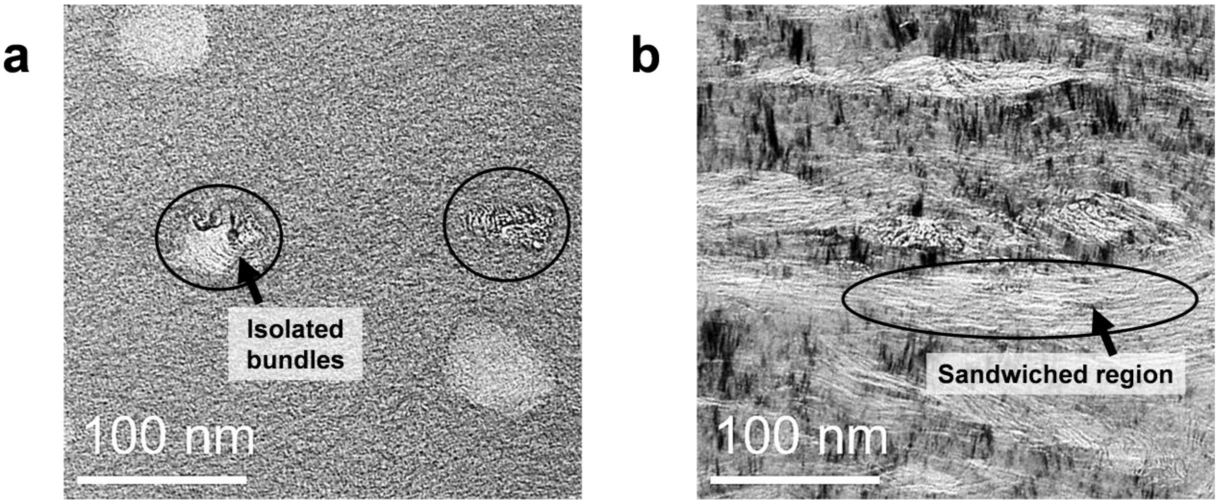

Figure S3. HR-TEM images of (a) B-CNT3-2000 and (b) B-CNT15-2000 films showing the isolated bundles and sandwiched interphase, respectively.

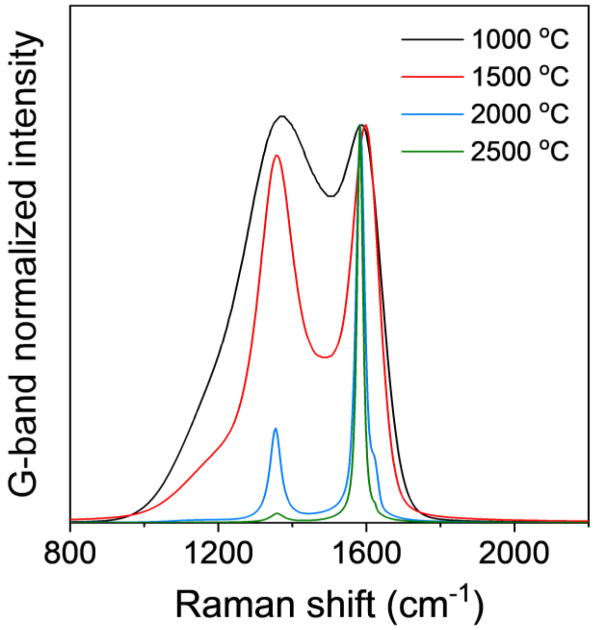

Figure S4. Raman spectra of GO15 films with different carbonization temperatures.

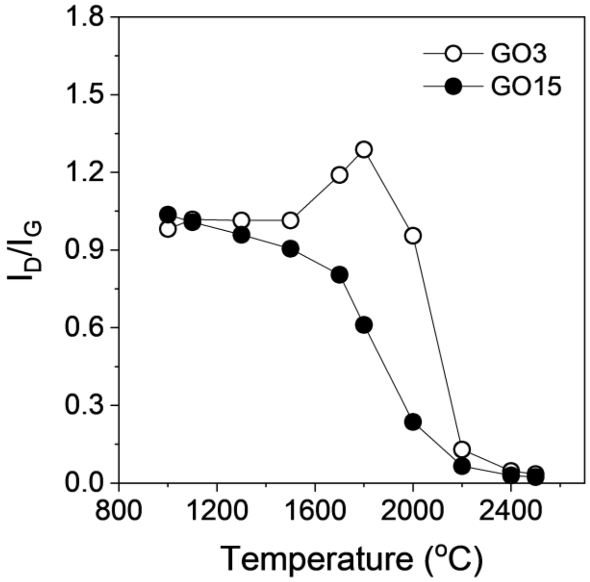

Figure S5. Changes in I_D/I_G ratio of GO3 and GO15 films with respect to the carbonization temperature.

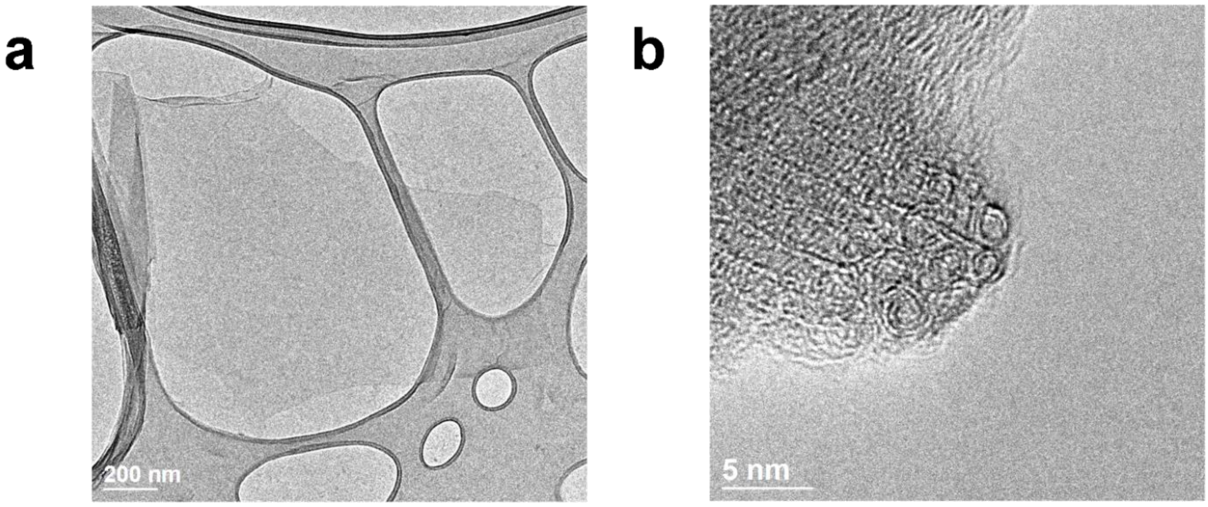

Figure S6. TEM images of dispersed (a) GO (lateral size: 0.58 ± 0.40 μm, thickness ~ 1 nm) and (b) SWNT (Diameter: 1.6 ± 0.4 nm, length > 5 μm) materials used in the current study.

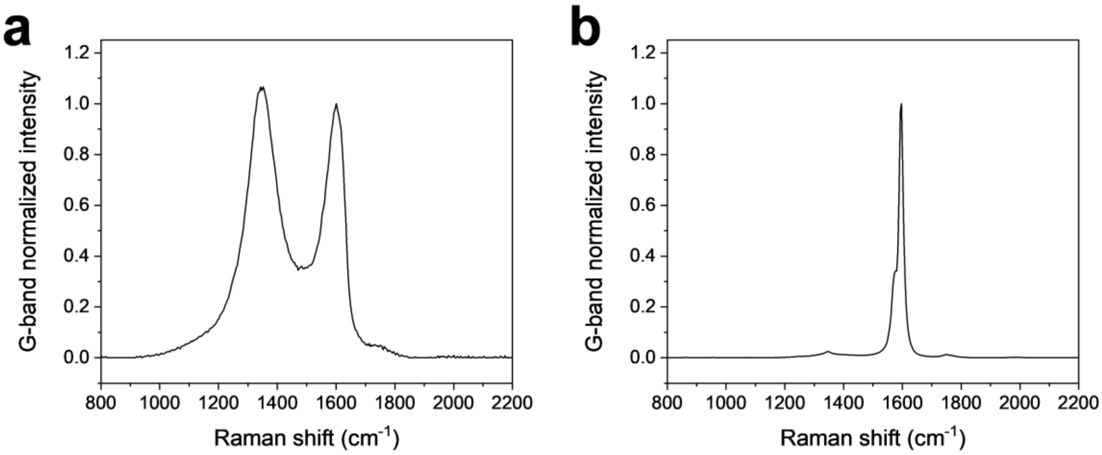

Figure S7. Raman spectra of (a) GO (I_D/I_G ~ 1.07 ± 0.02) and (b) SWNT (I_D/I_G ~ 0.02 ± 0.01) materials used in the current study.
